# Near-peer teaching of laparoscopic skills among medical students: a randomised feasibility study

**DOI:** 10.1186/s12909-025-08461-4

**Published:** 2026-02-21

**Authors:** Martin Patrick Ho, Leonie Heskin, Lena Dablouk, Yasmina Richa, Joshua Nolan, Andrew O’ Brien, Samin Abrar, Derek Hennessey

**Affiliations:** 1 Department of Surgery, University College Cork, Cork, Ireland; 2 ASSERT Research Centre, Brookfield Health Sciences Complex, University College Cork, Cork, Ireland; 3 Department of Urology, Mercy University Hospital, Cork, Ireland

**Keywords:** Near-peer teaching, Laparoscopy, Laparoscopic, Surgical skills, Surgical training, Medical students, Medical education, Simulation

## Abstract

**Background:**

Laparoscopic surgery is integral to modern surgical practice. However, many medical students graduate with limited exposure to essential skills. Near-peer teaching is a promising, student-led approach to address this gap, though its objective impact remains under-evaluated. The aims of this study were to develop a novel objective assessment tool, assess changes in students’ confidence and objective performance in laparoscopic tasks following near-peer versus self-directed training, and explore factors associated with performance.

**Methods:**

This single-blinded, randomised feasibility study enrolled 42 medical students without prior laparoscopic experience. Participants were randomly assigned to near-peer (Group 1) or self-taught group (Group 2) and completed six weekly sessions across four laparoscopic stations: Ball Transfer, Circle Cutting, Peg Transfer, and String. Performance was scored using a novel objective assessment tool developed through a Modified Delphi Method. Two blinded surgical trainees assessed all performances.

**Results:**

No significant differences were observed in pre-intervention objective performance between groups, except for the “Peg Transfer” station. Post-intervention, both groups improved significantly across all tasks (*p* < 0.001). Their confidence also improved (*p* < 0.001). The peer-taught group outperformed the self-directed group in “Circle Cutting” (*p* = 0.02), “String” (*p* = 0.01), and “Peg Transfer” station (*p* = 0.04). There was no relationship between age, gender, or video game experience and performance.

**Conclusions:**

This is the first study to demonstrate that near-peer teaching of laparoscopic skills improved laparoscopic performance among medical students compared with a control group. These findings support the integration of near-peer laparoscopic teaching into undergraduate surgical education. The novel assessment tool demonstrated sensitivity to change in performance pre- and post-intervention.

## Background

Early exposure to surgical skills is essential for those who wish to enter surgical training, as it facilitates baseline competency when entering the operating theatre. Furthermore, it may entice students who previously had not considered surgery as a career.

Despite this benefit, medical students receive little hands-on surgical training. To address this, many universities have adopted the “Near-peer” model to teach surgical skills [1, 2]. That is, senior or experienced medical students teach junior students basic surgical skills. This model addresses personnel limitations associated with limited surgical faculty. It also provides a low-cost, low-pressure, sustainable learning environment for students. Furthermore, this model allows students to develop collaborative leadership and teaching experience at a junior level.

Previous studies have evaluated near-peer teaching of suturing skills, however, only one study has examined laparoscopic skills, and only at a single station [3]. No study has comprehensively evaluated student performance across multiple laparoscopic stations. Laparoscopic surgery represents an increasing proportion of surgical procedures [4]. It possesses unique learning requirements, including spatial awareness, depth of perception, and dynamic force application.

Given these technical demands, reliably assessing performance is essential. Currently, no comprehensive, validated criteria exists to evaluate laparoscopic training performance. Previous metrics used include time to task completion [5]. However, trainees should prioritise correct surgical technique rather than speed. The Objective Structured Assessment of Technical Skills is a validated tool that was developed to evaluate surgical performance [6]. However, many of the questions are not applicable to laparoscopic training [3].

While several studies have evaluated the impact of a single or two-day surgical skills course [1, 2, 5], few have conducted an extended evaluation. Distributed practice over extended periods facilitates sustained skill improvement, which has been described as “a key marker of the efficacy of an intervention” [7].

Furthermore, few studies have assessed laparoscopic performance among medical students, a heterogeneous group with wide variation in psychomotor skills, making them an ideal population to study.

Although near-peer teaching of surgical skills is increasingly used in medical education, no study has rigorously evaluated sustained, objective improvements in student laparoscopic performance. This feasibility study had four aims: [1] to develop a novel objective assessment tool using the Modified Delphi Method; [2] to evaluate and assess change in student self-reported confidence; [3] to evaluate and assess change in student objective performance across multiple laparoscopic stations; and [4] to identify factors associated with objective performance.

## Methods

### Study design

This was a single blinded, prospective, randomised feasibility study. Ethical approval was obtained from the Clinical Research Ethics Committee of the Cork Teaching Hospitals (ECM 4(u) 19/11/2024 & ECM 3(e) 02/02/2025). Informed consent was obtained from study participants who approved use of their data for research purposes. While a formal power calculation was not possible, a pragmatic sample size of 42 participants was chosen to allow estimation of effect magnitude to inform future studies. This is consistent with recommendations that at least 30 participants be included in a feasibility study [8, 9].

### Stations

**Fig. 1:**
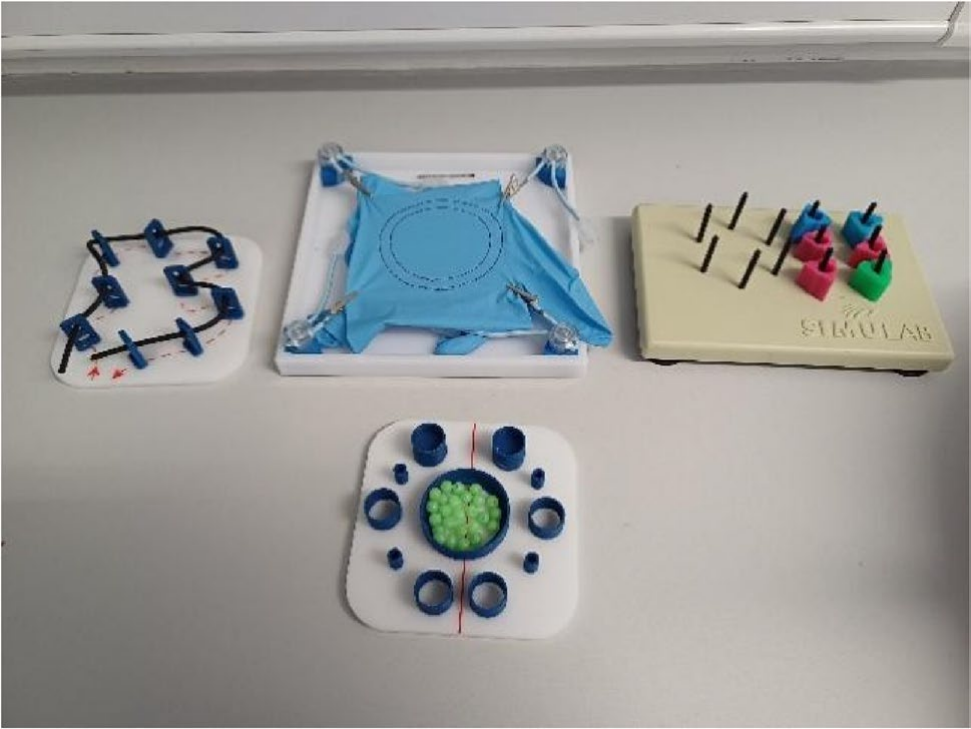
The four laparoscopic stations: string (top left), circle cutting (top centre), peg transfer (top right), ball transfer (bottom)

All participants used the Laparo^®^ Aspire Simulator [10] to complete four laparoscopic stations. These were Ball Transfer, Circle Cutting, Peg Transfer, and String, with time limits of seven, seven, two, and seven minutes respectively (Fig. 1). The “Peg Transfer” station trained foundational dexterity using bimanual co-ordination, midline transfer, and non-dominant hand use. As it was a simpler station, the time was reduced to two minutes. The “Ball Transfer” station was more difficult as it emphasized depth of perception and dynamic force application. This allowed for discrimination between scores. The “Circle Cutting” simulated tissue tension and replicated surgical dissection. It also allowed for strategies to be employed to complete the station. Finally, the “String” station tested depth of perception, anticipatory instrument positioning, and the ability to plan ahead.

### Laparoscopic training

All participants completed a pre-intervention questionnaire (Appendices 1, 2) and received a standardised video presentation, demonstrating the objectives of each station. Following this, participants completed a pre-intervention recorded assessment.

Next, participants completed 30 minutes of training. This was divided into four seven-minute stations plus one minute of rest in between. Altogether, each participant received three hours of training (six 30-minute sessions). Each training session was one week apart to evaluate for skill retention. Two “Catch-Up Sessions” were included, allowing those who had missed a training session to attend. Following the final training session, participants completed a post-intervention questionnaire and recorded assessment.

### Intervention

Prior to completing the pre-intervention questionnaire, participants were allocated to peer-taught (Group 1) or self-taught (Group 2) at a 1:1 ratio according to a random sequence generated by an independent administrator. Group 1 received instruction from senior medical students acting as near-peer teachers during training sessions, with a teacher-to-participant ratio of 1:4 to 1:5. Group 2 trained laparoscopy without near-peer teaching and were forbidden from teaching one another or from receiving feedback during the study period. Six near-peer teachers were recruited, each with at least six months of peer-teaching experience and prior completion of the Basic Surgical Skills Course [11]. Additionally, all near-peer teachers received a one-to-one tutorial from a surgical consultant covering the objectives of each station, ensuring correct technique was emphasised. They also completed the Professional Development Service for Teachers’ Team Teaching Module [12]. This online module was accessible and tailored to novice teachers, covering basic teaching theories, feedback, and roles of teachers within a large group.

### Modified Delphi method

A Modified Delphi Method was employed to develop an assessment tool for each station. It began with a draft questionnaire developed by the first author based on a literature review and preliminary framework. This was distributed to three experts in laparoscopic surgery and surgical education who provided feedback until unanimous consensus was achieved. The assessment domains of the assessment tool were unique for each station. Points were allocated for specific task anchors based on the performance quality of each step. The remaining points were allocated for bonuses, deductions, and subjective impression. The assessment tools are seen in Appendices 3–6.

### Statistical methods

Pre- and post-intervention recorded assessments were scored by two blinded surgical trainees and analysed. Participant identity and group allocation could not be determined from the recordings. Participant objective performance and subjective confidence were reported as median with interquartile range. Participant improvement was defined as the difference between the median pre-intervention and median post-intervention total scores according to the novel objective assessment tool. Spearman’s rank-order correlation and Mann Whitney U Test determined association between variables and objective performance. Where baseline parity was not achieved, an ANCOVA test was conducted after verifying the assumption of homogeneity of regression slopes. One-way Intraclass Correlation Coefficient was calculated for the two blinded reviewer scores. Analysis was performed with Jamovi version 2.6.26.

## Results

### Participants

Altogether, 42 participants were included. Mean participant age was 22.9 years. 14 (33.33%) participants were male, while 28 (66.67%) were female. Participant demographics are described in Table 1. Seven participants (16.67%) were lost to follow up.

**Table 1:** Participant demographics. participant age, gender, year of study, and previous video game experience, split by group

	Group 1	Group 2
*Age in Years (Mean +/- SD)*	22.9 +/- 4.33	22.8 +/- 3.04
*Gender*		
Male	8	6
Female	13	15
*Year of Study*		
Year 1	*11*	*9*
Year 2	*6*	*7*
Year 3	*3*	*4*
Year 4	*1*	*0*
Year 5	*0*	*1*
*Previous Video Game Experience*		
Yes	14	16
No	7	5

### Pre-intervention objective performance

Using the Mann Whitney U Test, there was no significant performance difference between Group 1 and Group 2 in the “Ball Transfer” (Median difference = 4, *p* = 0.37, Effect Size=−0.17), “Circle Cutting”, (Median difference = 0.5, *p* = 0.63, Effect size = 0.09), “String” station (Median difference = 3.7, *p* = 0.48, Effect Size = 0.14), or participant confidence. However, Group 1 outperformed Group 2 in the “Peg Transfer” station (Median difference = 12, *p* = 0.05, Effect size = 0.36). One-way Intraclass Correlation was 0.845 for the “Ball Transfer” station, 0.897 for the “Circle Cutting”, 0.951 for the “Peg Transfer”, and 0.846 for the “String” station. Data is shown in Table 2.

**Table 2:** Pre-intervention performance and confidence scores by group. Median scores for each station and self-reported confidence are shown for group 1 (peer taught) and group 2 (self-taught). Median differences between groups and corresponding p-values are reported

Station	Median Score Group 1	Median Score Group 2	Median Difference	Significance (*p*)
Ball Transfer	39	35	4	0.37
Circle Cutting	10	10.5	0.5	0.63
*Peg Transfer*	*16*	*4*	*12*	*0.05*
String	22.8	26.5	3.7	0.48
Confidence	10	10	0	0.83

### Post-Intervention improvement

**Fig. 2:**
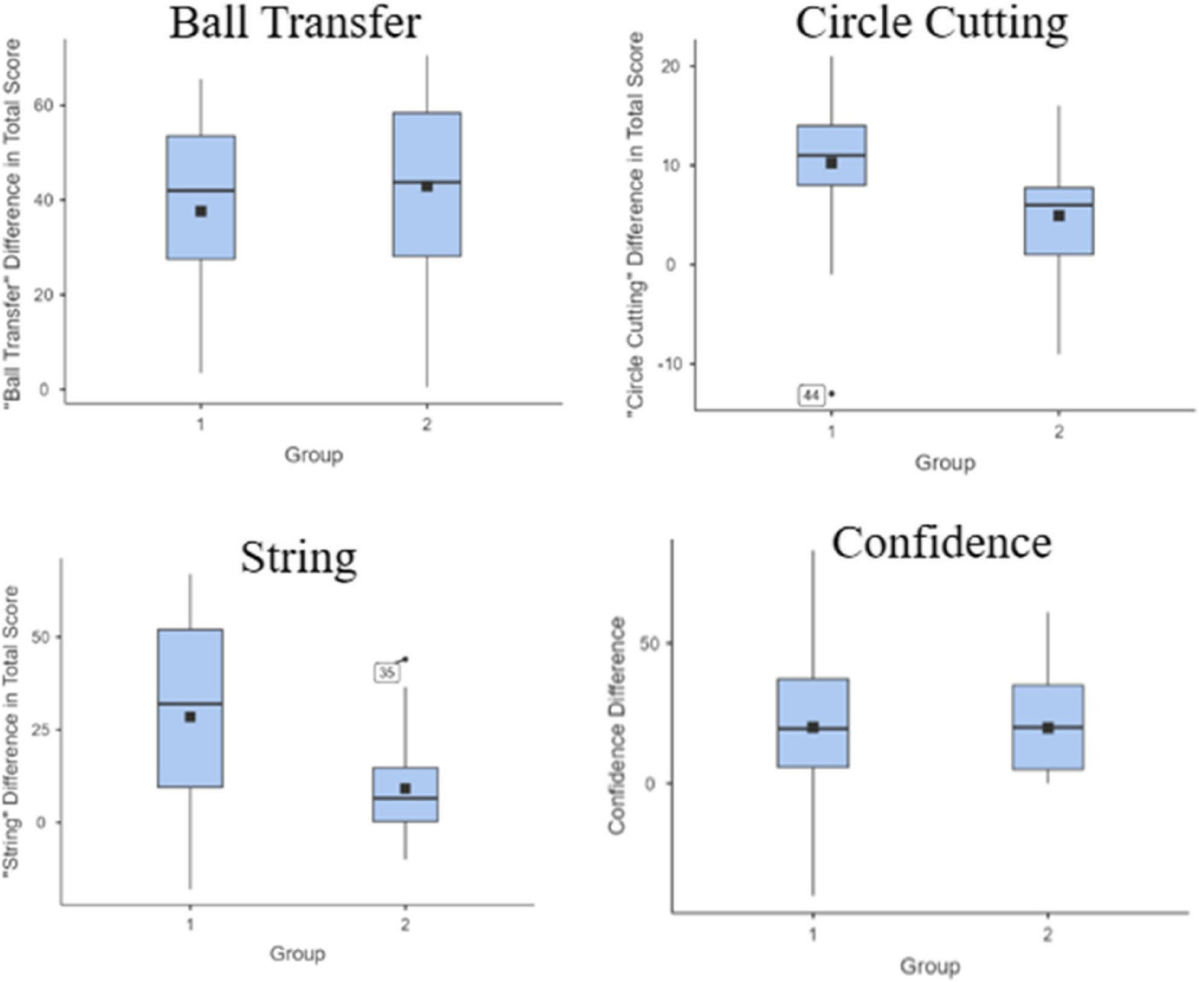
Box Plots of difference in pre- and post-intervention total scores for the “ball transfer”, “circle cutting”, “string” station, and participant “confidence”. the plots show median improvement in scores (central line), interquartile ranges (boxes), mean (dots), and outliers for participants in group 1 and 2

Post-intervention, participants in both groups improved across all four laparoscopic stations. The results for each station are summarised in Table 3. Using Wilcoxon Signed Rank Test, there was a significant improvement in both groups’ post-intervention objective scores in “Ball Transfer” station (W = 0, *p* < 0.001). No significant difference in improvement was seen between the two groups (*p* = 0.77) (Fig. 2). Similarly, both groups improved significantly in the “Circle Cutting” station (W = 34.5, *p* < 0.001). Group 1 outperformed Group 2 by five points (*p* = 0.02, Effect size=−0.44) (Fig. 2). Additionally, both groups improved significantly in the “String” station (W = 27.5, *p* < 0.001). Group 1 outperformed Group 2 by 25.5 points (*p* = 0.01, Effect size=−0.49) (Fig. 2). Additionally, both groups’ confidence improved significantly following the intervention (W = 16.5, *p* < 0.001), with no significant difference in improvement in confidence between the two groups (*p* = 0.44) (Fig. 2).

**Table 3:** Improvement in performance and confidence scores by group. Median scores for each station and self-reported confidence are shown for group 1 (near-peer taught) and group 2 (self-taught). Median differences between groups and corresponding p-values are reported

Station	Median Improvement Group 1	Median Improvement Group 2	Median Difference	Significance (*p*)
Ball Transfer	42	43.8	1.8	0.77
*Circle Cutting*	*11*	*6*	*5*	*0.02*
*String*	*32*	*6.5*	*25.5*	*0.01*
Confidence	19.5	20	0.5	0.44

**Fig. 3:**
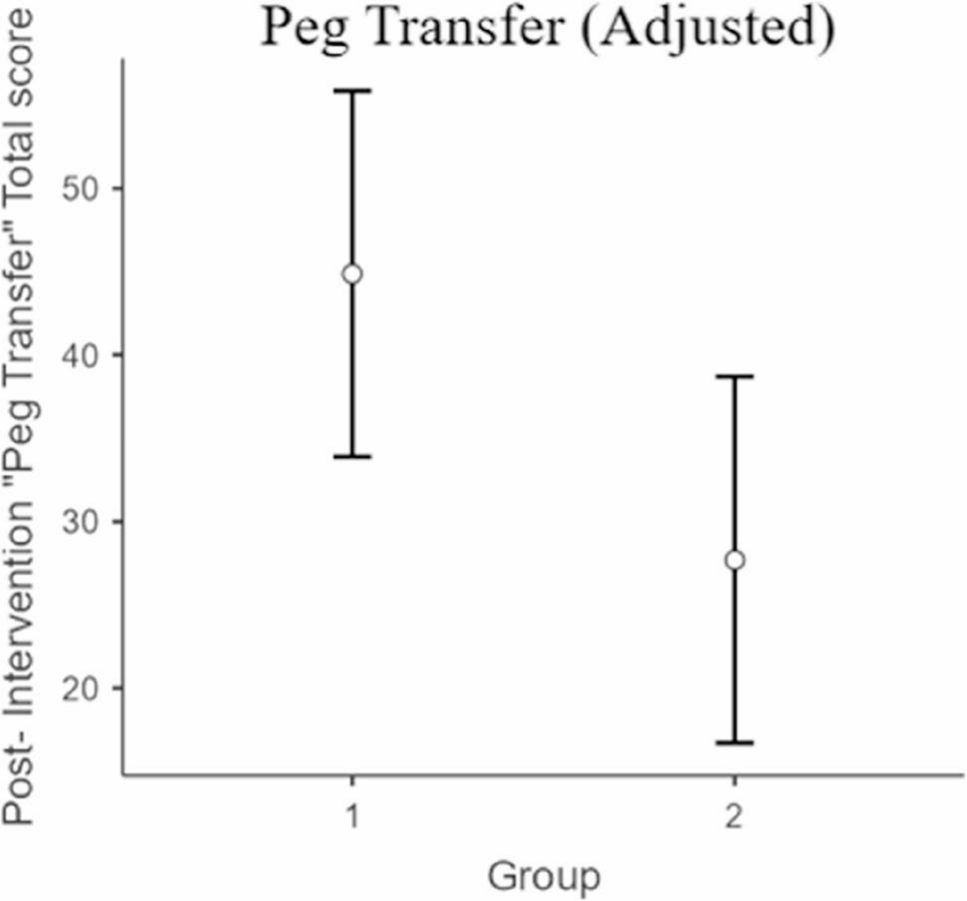
Adjusted post-intervention “Peg Transfer” Total Scores by Group Estimated marginal means (± 95% confidence intervals) for post-intervention “Peg Transfer” Total scores derived from the ANCOVA model, controlling for baseline (pre-intervention) performance. Group 1 demonstrated higher adjusted post-test performance compared with group 2

Both groups improved significantly in the “Peg Transfer” station (W = 29, *p* < 0.001). After controlling for baseline performance, the effect of group on improved objective performance was statistically significant, (F(1, 29) = 4.64, *p* = 0.04) (Table 4). Group 1 showed higher adjusted post-intervention “Peg Transfer” scores (EMM = 44.39, 95% CI, 33.9–55.9) compared with Group 2 (EMM = 27.7, 95% CI, 16.7–38.7) (Fig. 3).

**Table 4:** Analysis of covariance for post-intervention “Peg transfer” score. Results of the ANCOVA examining group differences in post-intervention “Peg Transfer” performance scores while controlling for baseline (pre-intervention) “Peg Transfer” performance scores. The model includes group as the fixed factor and pre-intervention objective performance as the covariate

	Sum of Squares	df	Mean Square	F	*p*
Overall model	1945.3	2	972.6	3.0674	0.062
Group	1930.3	1	1930.3	4.6382	*0.040*
Pre “Peg Transfer” Total score	14.9	1	14.9	0.0358	0.851
Residuals	12069.3	29	416.2		

### Factor analysis: objective performance

Pre-intervention confidence was weakly correlated with “Ball Transfer” (*r*=−0.24), “Circle Cutting” (*r* = 0.02), “Peg Transfer” (*r* = 0.1), and “String” performance (*r*=−0.22). Participant age, gender, previous interest in surgery as a career, and video game exposure were not significantly associated with performance in any stations.

## Discussion

This study represents the first comprehensive evaluation of near-peer teaching of laparoscopic surgical skills among medical students. The results suggests that near-peer teaching of laparoscopic skills improves objective performance, compared with self-teaching.

Our first aim was to develop a novel objective assessment tool using the Modified Delphi Method. Our objective assessment tool successfully demonstrated improvement between pre- and post-intervention performance and between groups. Once validated, this tool offers medical students an accessible, objective metric to evaluate their laparoscopic performance.

In addressing our second aim of assessing change in student self-reported confidence, we observed that participants’ confidence in their surgical skills improved following practice, independent of group allocation. This is consistent with previous literature [13, 14]. While a systematic review has characterised subjective confidence as a “poor predictor” of performance [7], it may serve as a motivator to continue practising, and develop interest in surgery, as described in the competence-motivation theory [15, 16]. Therefore, the use of confidence as an outcome measure may be beneficial within undergraduate surgical education.

We also achieved our third aim of evaluating student objective laparoscopic performance. Participant pre-intervention objective performance was measured to establish parity between groups. No significant differences were observed between groups across the stations, except for the “Peg Transfer” task, where Group 1 performed better. This isolated finding was likely due to chance, however it prompted additional statistical analysis to evaluate post-intervention improvement. Although analysis of covariance yielded statistically significant results, this baseline disparity may limit the internal validity of results for this station.

Both groups improved significantly in all four stations post-intervention. This is the first study to demonstrate improved objective laparoscopic performance. Currently, many surgical societies lack laparoscopic trainers due to financial constraints [17]. The improvement in objective performance underscores the importance of funding and investment in laparoscopic equipment.

Group 1 significantly outperformed Group 2 in the “Circle Cutting” station, “String” station, and “Peg Transfer” station. These findings suggest that near-peer teaching positively influences objective laparoscopic performance, consistent with previous studies [18, 19]. The concept of cognitive and social congruence underpins near-peer teaching; It suggests that learners better understand material when taught by peers, as they share a similar knowledge foundation and feel more comfortable and supported [15].

However, Group 1 did not outperform Group 2 in the “Ball Transfer” station (*p* = 0.77). This may be due to station design, as it emphasised dynamic force manipulation which may be difficult to verbally explain and teach. This aligns with theory of cognitive load [20]; a technically complex and unstable station may overwhelm learners’ working memory, negating the advantage of external instruction.

Our final aim was to identify factors associated with objective laparoscopic performance. We noted that patient age, previous interest in surgery as a career, and video game exposure were not associated with improved objective performance in any station. This contrasted with previous studies’ results [21, 22]. While our limited sample size may limit external validity of our results, they question the assumption that younger, technology-experienced individuals are inherently better at laparoscopic surgery. Our results suggest that modifiable factors, such as time spent practising may be more significant determinants of skill acquisition. This highlights the benefit of near-peer teaching and providing early surgical skills training opportunities to medical students.

This feasibility study employed a randomised design with a control group and two blinded evaluators. Despite many medical students attending out-of-city hospital placements, only seven (16.67%) participants were lost to follow up. An attrition rate below 20% is considered to pose minimal risk to study validity [23]. Furthermore, our attrition rate is comparable to previous studies with only a two-session workshop [18]. This may reflect our inclusion of two additional “Catch-Up Sessions” to maximise participant completion.

Limitations associated with the study include its single centre design and limited sample size with convenience sampling. This ensured feasibility, however limited the external validity of results and ability to draw firm conclusions that inform educational practise.

Regarding the “Peg Transfer” station, although analysis of covariance adjusted for baseline differences, this approach cannot completely eliminate the influence of initial group imbalance. Residual confounding may remain and the adjusted group differences should be interpreted with caution for this station.

Additionally, while the novel assessment tool proved sensitive to change in performance, it was not externally validated. This limited the validity and comparability of results.

## Conclusions

We successfully developed a novel objective assessment tool to evaluate student objective laparoscopic performance. Near-peer teaching significantly improved laparoscopic performance among medical students compared with self-directed learning. We also observed increased student confidence in their surgical skills. These findings support the inclusion of near-peer teaching within undergraduate curricula. A multi-centre randomised controlled trial, powered using effect sizes generated from this study is warranted to confirm efficacy and validate the novel assessment tool.

## Appendix

### Participant questionnaire page 1 of 2


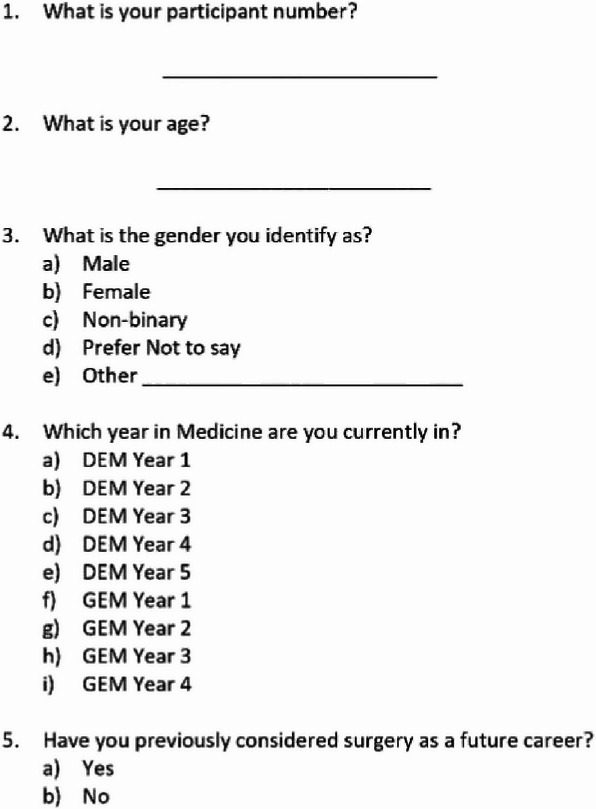


### Participant questionnaire page 2 of 2


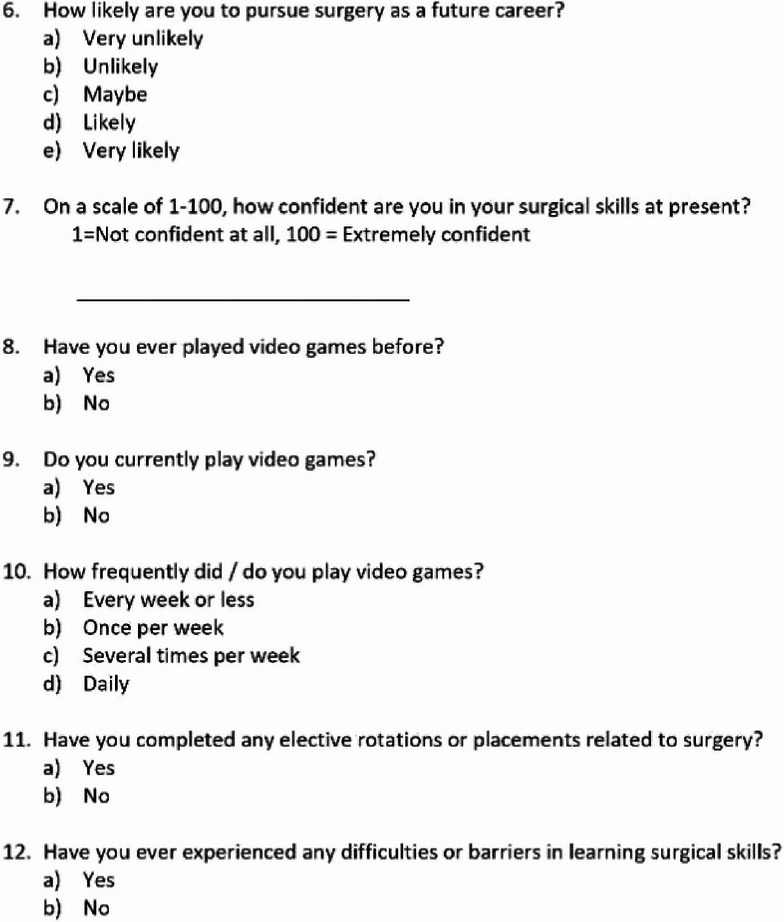


### Ball transfer evaluator sheet


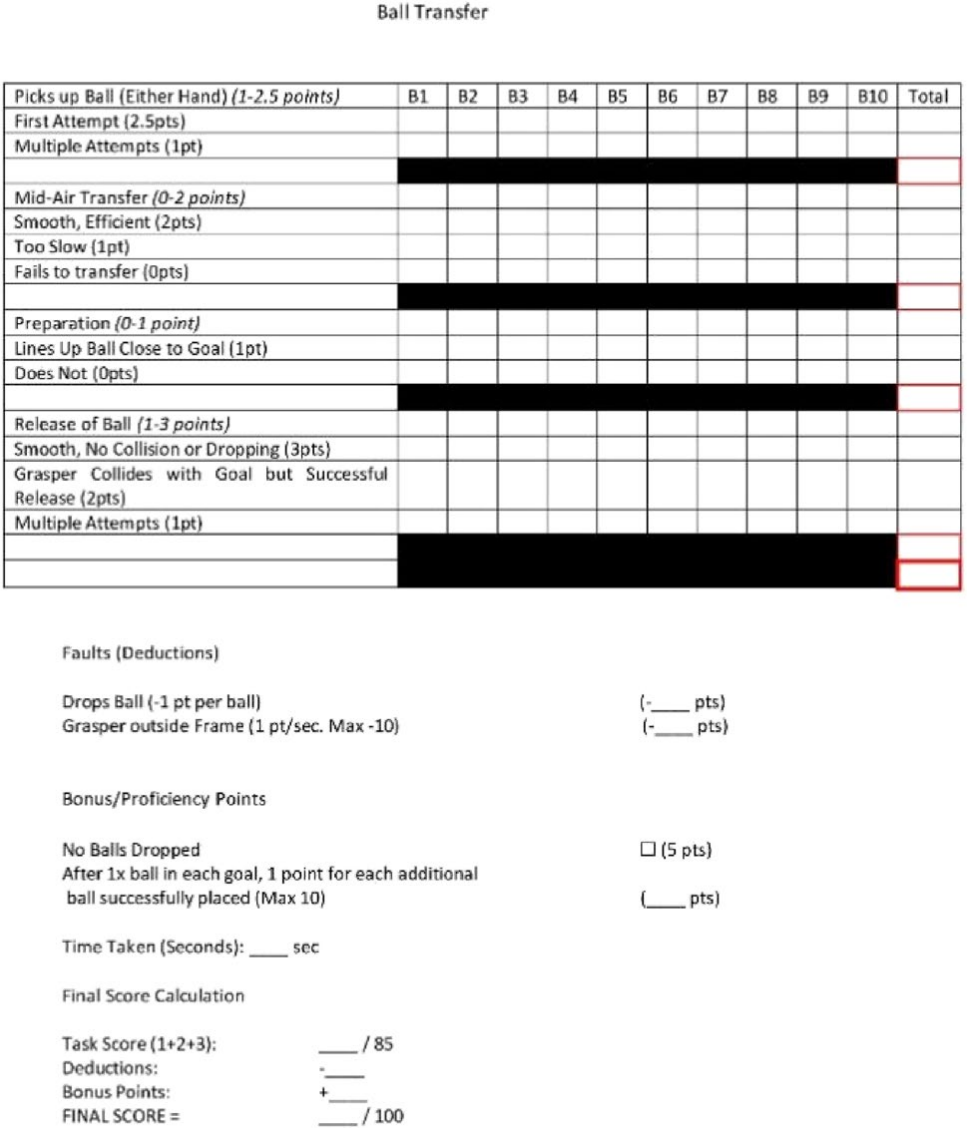


### Circle cutting evaluator sheet


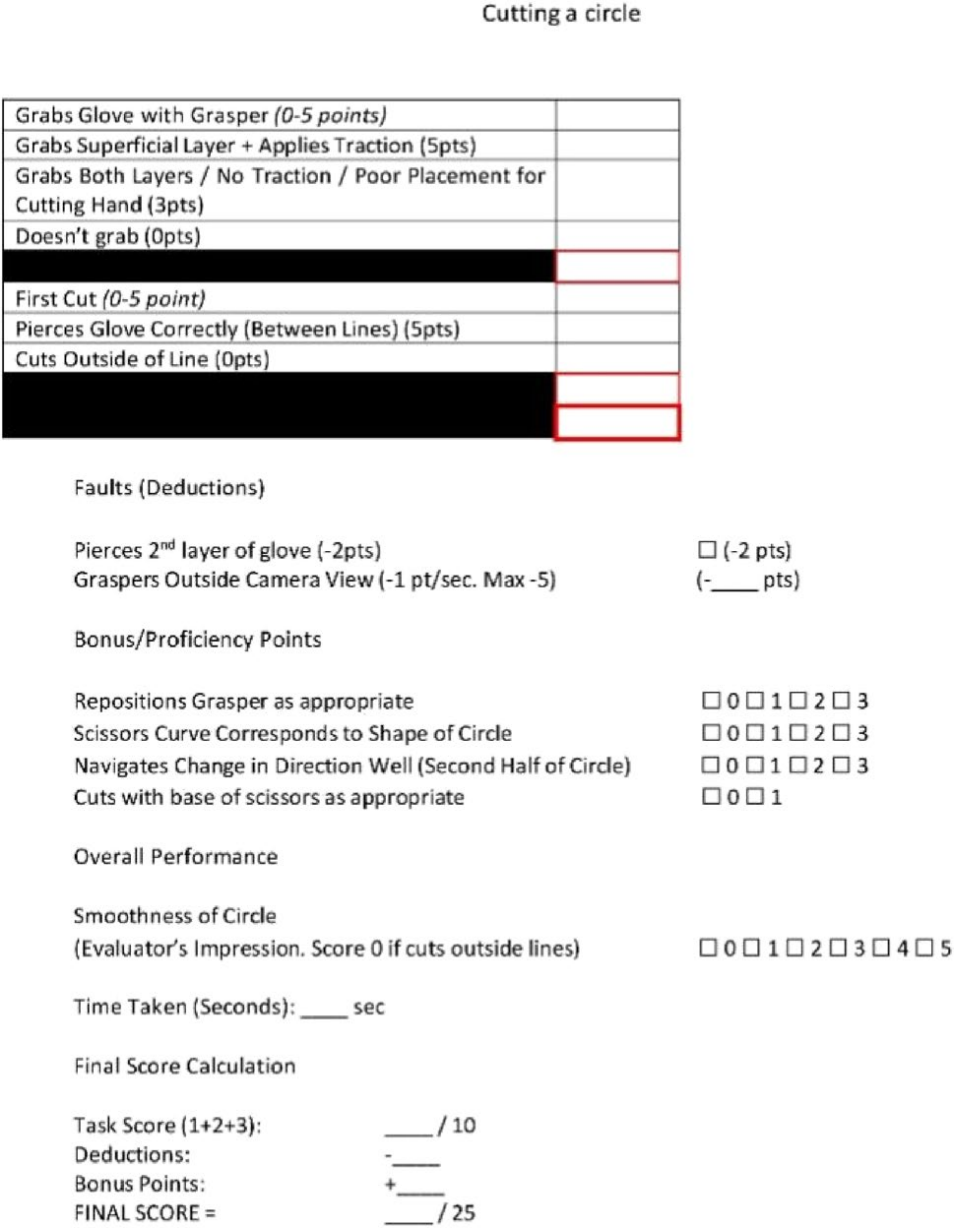


### Peg transfer evaluator sheet


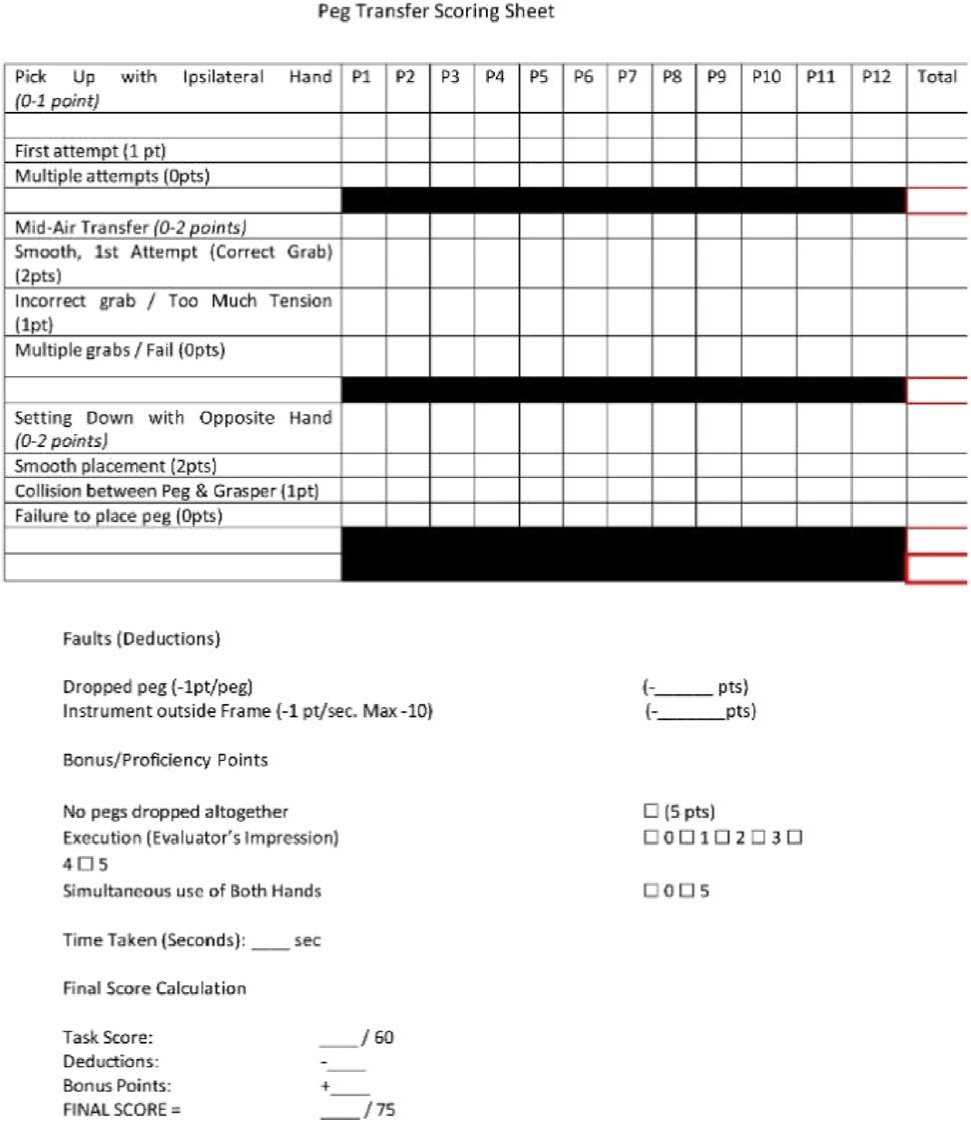


### String evaluator sheet


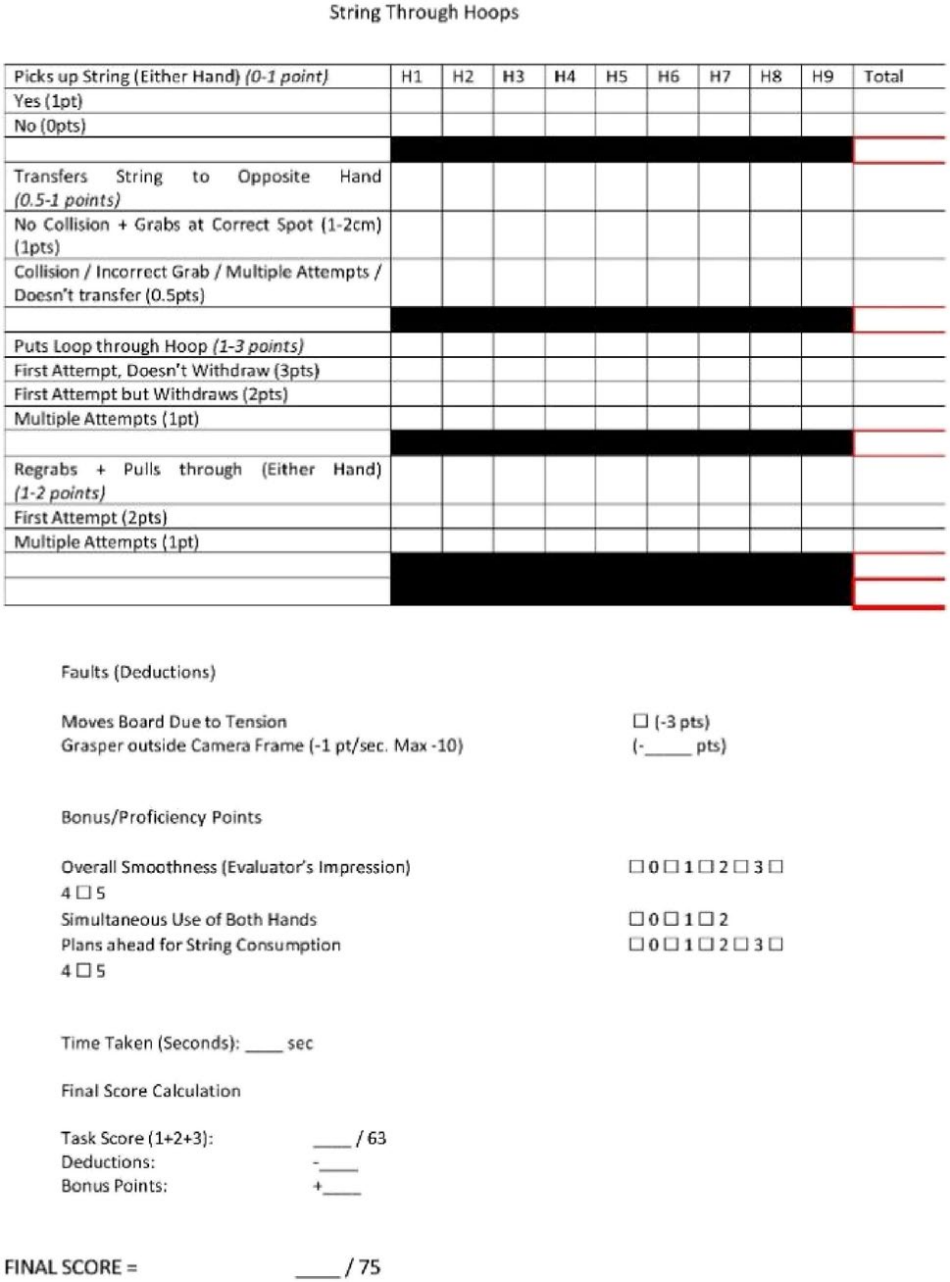


## Data Availability

Participants provided informed consent for the use of their data for research purposes, including publication, but did not consent to public data sharing. In accordance with the approval granted by the Clinical Research Ethics Committee of the Cork Teaching Hospitals (ECM 4(u) 19/11/2024 & ECM 3(e) 02/02/2025), data may be made available upon reasonable request to the corresponding author, subject to ethical and institutional approval.
